# Antibiotic Prophylaxis on Third Molar Extraction: Systematic Review of Recent Data

**DOI:** 10.3390/antibiotics8020053

**Published:** 2019-05-02

**Authors:** Gabriele Cervino, Marco Cicciù, Antonio Biondi, Salvatore Bocchieri, Alan Scott Herford, Luigi Laino, Luca Fiorillo

**Affiliations:** 1 Department of Biomedical and Dental Sciences, Morphological and Functional Images, School of Dentistry, University of Messina, 98100 Messina, Italy; gcervino@unime.it (G.C.); salvo.bocchieri@gmail.com (S.B.); lfiorillo@unime.it (L.F.); 2 Department of General Surgery and Medical Surgery Specialties, University of Catania, 95100 Catania, Italy; abiondi@unict.it; 3 Department of Maxillofacial Surgery, Loma Linda University, Loma Linda, CA 92354, USA; aherford@llu.edu; 4 Multidisciplinary Department of Medical-Surgical and Odontostomatological Specialties, University of Campania “Luigi Vanvitelli”, 80121 Naples, Italy; luigi.laino@unicampania.it

**Keywords:** third molar, oral surgery, extraction, antibiotic, prophylaxis

## Abstract

The aim of this paper was to highlight the most widely antibiotic protocols applied to the dental field, especially in the surgical treatment of impacted wisdom teeth. Once these protocols were screened, all the possible advantages or disadvantages for each drug and each posology were recorded in this review. In recent years, the need to use these protocols has been debated in the literature. The data obtained by this review underlined how antibiotic protocols applied to oral surgery treatments only included surgeries performed on patients who did not present other systemic pathologies. The first literature review obtained 140 results, and then after the application of the inclusion criteria, 12 papers were selected. The results showed that the most commonly used protocol involved the use of penicillin and clavulanate, obtaining safe clinical and prophylactic results in the management of infections. This widely used protocol seems to guarantee high predictability and safety. The presented review highlights the current possibility of antibiotic resistance affecting patients due to drug misuse. Further clinical studies are required to state specific guidelines; however, oral surgeons involved in third molar surgery should evaluate the local and general health conditions of the patients before suggesting any drug measures for patients.

## 1. Introduction

The surgical avulsion of wisdom teeth has now become a common surgical practice. However, it is a surgical practice that can expose patients to possible intra and post-operative infection [1]. The antibiotic protocols used during this surgery are different. Clinicians should always evaluate the general conditions of the patients and the presence of any allergies or intolerances. In recent years, the need to undertake antibiotic prophylaxis in patients undergoing this type of surgery has been much debated, assessing the costs/benefits. In addition to systemic antibiotic prophylaxis, topical disinfection of the operating field is usually performed during the operation, and topical home therapies are prescribed to be applied subsequently [2]. However, the risk of bacterial infection of the surgical field is always possible. The ultimate goal of this work is to understand which antibiotic protocols are most used during third molar surgery and then evaluate their advantages and disadvantages. This article therefore has the function of showing all the protocols but highlighting which are the best to use based on the patient’s clinical and surgical condition [3,4].

The prescription of systemic antibiotics for the prevention of complications such as alveolitis and infection of the surgical site during the extraction of the third molar is a widespread practice among dentists, but this is also controversial and debated. The controversy arises because prophylactic antibiotic therapy is usually not indicated in healthy patients, and inappropriate use of antibiotics puts patients at risk of adverse reactions and contributes to the development of antibiotic resistance. Furthermore, considering the cost–benefit ratio, systematic reviews and published meta-analyses do not support the routine prophylactic use of antibiotics. In any case, patients undergoing this surgical therapy, in addition to antibiotic prophylaxis, are often forced to use other pharmacological therapies based on the duration and the complexity of the intervention for the management of the post-operative phase [5,6,7,8,9]. The eruption of the four wisdom teeth establishes the completion of permanent dentition: in normal conditions, each wisdom tooth occupies the last position of each dental semi-arch. However, the third molars do not always erupt: it is not rare that one or more wisdom teeth remain embedded in the bone and gum. In such circumstances, the incomplete permanent dentition reflects a condition known as hypodontia (fewer than four wisdom teeth). At other times, even though they partially succeed in erupting from the gingiva, wisdom teeth do not complete their development; in such circumstances, the third molars, not finding sufficient space to complete growth, remain anchored in the maxillary or mandibular bone. The incomplete development of wisdom teeth can predispose to the formation of chronic inflammatory foci, making it necessary to extract the tooth. The presence of this inflammatory focus can lead in other cases to the formation of osteolytic lesions caused by the involved tooth [10,11,12].

The purpose of this systematic review of the literature is therefore to highlight which antibiotic protocols are most used during wisdom tooth surgery, and to evaluate among these the best therapeutic, pharmacological, and posological routes for the patient, limiting the misuse of antibiotic drugs where possible.

## 2. Results

### 2.1. Manuscript Collection and Search Strategy

**Figure 1:**
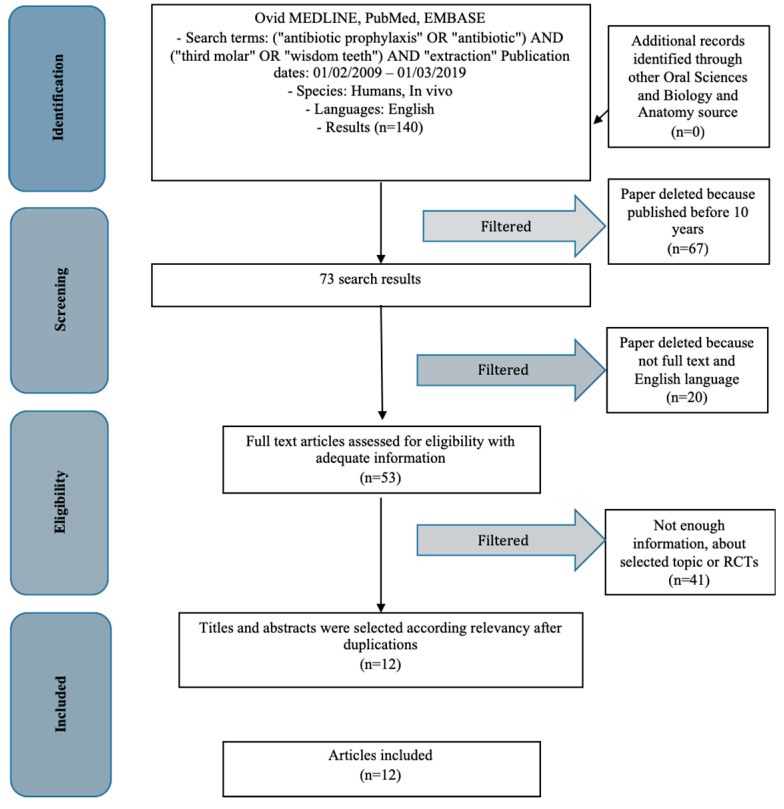
PRISMA (Preferred Reporting Items for Systematic Reviews and Meta- Analyses) flow diagram. RCT: randomized controlled trial.

A literature search was conducted and gave a high number of results: 140. Subsequently, filters were applied to make the research and results more specific. Firstly, studies from the last 10 years were evaluated (73 studies; on humans 53), then those that had accessible full texts (to be able to analyze the results), randomized clinical trials (RCTs), and then those in English. Only 12 studies were included in this review (Figure 1). The keywords used to search the databases were as follows: (“antibiotic prophylaxis” OR “antibiotic”) AND (“third molar” OR “wisdom teeth”) AND “extraction”.

The choice of keywords was intended to produce as many results as possible to support the review. A manual search was also conducted on textbooks to increase the scientific support and accuracy of the study. The textbook research did not produce sources in support of the review but provided information regarding the introduction and discussion sections.

### 2.2. Study Characteristics

The results obtained subsequently were categorized based on the dosage, type of drug, and type of extraction surgery performed. The studies taken into consideration by this revision were all RCTs. Each article presents information about the use of antibiotic protocols during wisdom tooth extraction surgery. The information is shown in Table 1.

### 2.3. Risk of Bias Within the Studies

Evaluation on the total risk of bias for each selected paper, and the majority of the manuscripts were allocated as low risk (Table 2) [13,14,15,16,17,18,19,20,21,22,23,24]. Studies evaluated were all RCTs using the double blinded method. A “low risk” study uses a valid approach to allocate patients to alternative treatments and results are considered valid. A “moderate risk” or “medium” study is susceptible to some bias but probably not enough to invalidate the results and may be missing information. A “high risk” rating indicates significant bias that may invalidate the results. In this case there are large amounts of missing information or discrepancies in reporting.

### 2.4. Risk of Bias Across the Studies

Numerous limitations have arisen from the present revision. Current analysis of the data extracted from studies written in English only could introduce a possible publication bias. The main limitation of the revision is related to the oral surgery and to the third molar surgery. The use of antibiotics is still debated widely.

### 2.5. Evaluation of Studies

The statistical analysis of the studies was singularly analyzed. The authors disagree on different parameters evaluated after surgery. Some studies take into consideration the duration of surgery, which was not evaluated in this study and is not presented in the tables; this seems to have a fundamental importance in the management of complications. On other topics the authors disagree, but most of them show that pain, swelling, fever, edema, reduced mouth opening, or postoperative surgical site infection do not show significant differences between study groups. Therefore, we have no significant statistical differences between the different types of antibiotics and between antibiotic and placebo, leading to the conclusion that performing surgery of wisdom teeth in the absence of antibiotic prophylaxis does not lead to more difficult management of complications.

## 3. Discussion

### 3.1. Context of Extractive Surgery

Surgical avulsion of third molars or wisdom teeth, as can be seen from the results, is a surgical procedure that often requires an antibiotic protocol by the clinician. However, some studies show that it is not always necessary [13,14,15,16,17,18,19,20,21,22,23,24]. The pharmacological protocols that are carried out during the surgery of the wisdom tooth do not only concern antibiotic prophylaxis. The management of post-operative or pre-operative pain in the case of acute inflammation certainly occurs through the use of correct analgesic and anti-inflammatory therapy. Both cyclooxygenase inhibitors 1,2 (NSAIDs), cyclooxygenase 3, and corticosteroid drugs are used for pain management. Certainly, salicylates drugs are not recommended in order not to expose the patient to their anti-aggregation action. Besides pain management, some drugs are suitable for the management of the intraoperative patient. For example, drugs to manage anxiety (anxiolytics) allow the operator to perform surgery with better patient compliance.

Regarding the topical use of drugs, we must not forget local anesthetics with and without adrenaline. Topical disinfection of the operating field often occurs with chlorhexidine-based mouthwashes or povidone iodide [25,26]. It is always advisable during the surgery of the octaves to follow the rules of oral surgery and periodontology in the management of hard and soft tissues. It is necessary to go to the distal bone peak at the lower second molar and allow a correct closure of the flaps when possible, limiting the risk of infection, dehiscence, or even bone exposure. When the tooth is regularly positioned in the jaws and therefore is not an impacted wisdom tooth, the surgical site can have second intention healing, using only hemostatic maneuvers and possibly sutures to contain the clot [27]. Bone remodeling has a good potential for growth as the distal bone peak is represented by the branch of the mandible.

During these surgeries it is possible to use different topical hemostatic procedures or even medicated gauzes, especially in the case where annexed osteolytic lesions are present. Among the hemostatic practices it is possible to recognize the use of collagen sponges; it is certainly not necessary to proceed with bone regenerative maneuvers [28], but a regularization of the post-extractive crest will suffice. Indeed, the mandibular branch and the retromolar region often represent being sites of autologous bone graft [29,30]. As with any other surgery it is necessary to carry out a correct anamnesis of the patient in order to highlight relative or total contraindications to oral surgery or even local or systemic contraindications. Certainly the patient’s clinical conditions take on great importance, and some common conditions, such as diabetes [31], may have important systemic implications, representing in some cases an absolute contraindication to treatment. Therefore, unfavorable clinical conditions may represent an absolute contraindication unless dental surgery is necessary to remove all oral inflammatory foci [32,33,34]. The complications of this surgery as already mentioned in the previous sections may be different and more or less long term. It is possible to use instruments that allow the clinician to be more conservative and more respectful towards the tissues, especially with respect to the anatomical structures [35,36,37,38].

### 3.2. Review Study Discussion

In the article of Monaco et al. [13], they evaluated 59 medically healthy patients subdivided into two groups: the amoxicillin 2 g and no antibiotics group (control group). All patients were undergoing dental surgery due to orthodontic reasons. Postoperative complications, like pain, swelling, or fever were evaluated and are shown in Table 3, with significant differences in some complications. In the 145-patient study of Luaces-Rey [14] there was no statistical difference between them. Siddiqi et al. [15] in their article reported that some postoperative complications were evaluable in the placebo control group in a study with a total of 100 patients. In the 800-patient sample in the study of Bezerra et al. [16], two groups (amoxicillin vs. placebo) were evaluated during third molar extraction. Differences in the frequency of inflammatory/infectious events was not observed between the experimental and control groups when osteotomy and tooth sectioning were performed. Adde et al. [17], in a different study, evaluated two different antibiotics, amoxicillin and clindamycin, versus placebo. In this case there were no statistical differences. However, they specified that all surgeries were performed after a topical disinfection with chlorhexidine. Sisalli et al. [18] conducted an Italian study in 2012 with the aim of comparing the effectiveness and the side effects of two different drugs, amoxicillin and clavulanic acid vs. ceftazidime, used as antibiotic prophylaxis in the surgical extraction of third molars. In this study there were 107 patients and two groups, amoxicillin vs. ceftazidime. According to Sisalli et al., the results showed that there was no statistical difference between the groups about complications after surgery. Duvall et al., in an RCT, evaluated the prevalence of bacteremia after third molar surgery [19]. In this study they evaluated three groups with placebo rinse and placebo pills, placebo rinse and amoxicillin, and placebo pills and chlorhexidine (0.12%). According to this study there was no statistical differences between each 10-person group. Crincoli et al. [20], in the Bari School of Dentistry, enrolled 24 patients for their study. The analysis of the data showed that oral and intramuscular antibiotic therapies almost overlap in preventing postoperative complications in dental surgery. However, the higher cost and the discomfort of the patient do not justify routine intramuscular antibiotic therapy, and should be reserved for patients with gastrointestinal disorders. In a double-blinded RCT, Arteagooitia et al. [21] evaluated the use of amoxicillin versus placebo during third molar extraction. In this study the same surgeon performed all extractions in a blinded way. They evaluated some postoperative parameters and they did not show significant differences. In another study by Milani et al. [22], they evaluated two different protocols using amoxicillin and a placebo group. They reported that despite the controversy surrounding antibiotic therapy in third molar surgery, systematic reviews are unanimous in concluding that more well-defined, controlled randomized trials are needed on the subject. Also in this study, there was no advantage found with the use of antibiotics for this type of surgery. Xue et al. [23] in a double blinded study evaluated systemic and local compliance after molar extraction. They made a classification based on the difficulty of the surgery according to the Pell–Gregory third molar classification [39]. There was no significant difference between antibiotic and placebo group during healing phases. Pol et al. [40] evaluated the use of laser therapy reducing pain and inflammation levels to third molar area; this is a useful tool in oral surgery as in other dental fields to treat soft and hard tissue [41]. Braimah et al. [24] in an observational study evaluated three different protocols using amoxicillin or levofloxacin. Single bolus amoxicillin or levofloxacin were less efficient for managing postoperative sequelae on extended amoxicillin prophylaxis. According to some systematic reviews like that of Marghalani et al. [42], the use of antibiotics can reduce the infection or dry alveolitis risk. However, antibiotic use can result in some systemic adverse effects to patients. Unfortunately, from many studies taken into consideration in this review, it is not possible to go back to the place where the RCTs were carried out. Specifically, it is not even possible to obtain information on the type of environment where the trials were conducted. This certainly appears to be a major limitation for the work.

## 4. Materials and Methods

### 4.1. Application Protocol and Website Recording Data

A protocol including the investigation methods and the inclusion criteria for the current revision was submitted in the PROSPERO website, an international prospective register of systematic reviews. The parameters and the analytic structure of the present work can be visualized relating the CRD ID and code; this systematic review was submitted at the PROSPERO website platform, with PROSPERO acknowledgement of receipt number 131364.

The data of this systematic investigation observed the Preferred Reporting Items for Systematic Review in accordance with the PRISMA statement.

### 4.2. Target Questions

The questions processed the following guidelines, according to PICO (P – patient, problem or population. I – intervention. C – comparison, control or comparator. O – outcome):

- What are the most widely used antibiotic protocols during wisdom tooth extraction surgery?
- Are there any alternatives? Are these more beneficial for the patient?

### 4.3. Search Strategy

We conducted a search in five electronic databases, including Ovid MEDLINE, PubMed, and EMBASE. In addition a manual search on Dentistry and Pharmacological source was conducted, for relevant studies published.

Digital and searches by hand were then performed in third molar extraction and antibiotic. In-depth research of the reference lists in the recorded manuscripts was performed in order to add significant studies and to increase the sensitivity of the revision.

### 4.4. Collection Data

Medical Subject Headings (MeSH) were applied for finding the keywords used in the present revision. The selected key words: (“antibiotic prophylaxis” OR “antibiotic”) AND (“third molar” OR “wisdom teeth”) AND “extraction” were recorded for collecting the data. The date of last search with these results was 31 March 2019.

### 4.5. Manuscript Selections

Two independent reviewers of two different universities (Messina and Naples) singularly analyzed the obtained papers in order to select inclusion and exclusion criteria as follows. Reviewers correlated their evaluations and analyzed differences through comparing the manuscripts and consulting a third experienced senior independent reviewer (H.A.S.; University of Loma Linda) when a consensus could not be reached. For the stage of the full-text articles revision, a complete independent dual analysis was performed.

### 4.6. Research Classifications

The method of classification included all human prospective and retrospective clinical studies, split mouth cohort studies, case–control papers, case series manuscripts, animal investigations, and literature reviews published between February 2009 and March 2019, on antibiotics used for oral surgery and third molar extraction.

### 4.7. Exclusion and Inclusion Criteria

The full texts of all studies related to the main revision topics were obtained for comparing the inclusion parameters:

- Investigated pharmacological prophylaxis or third molar extraction
- Clinical human randomized controlled trials

The following exclusion criteria:

- Patients with other specific disease as osteoporosis, immunologic disorders, uncontrolled diabetes mellitus, or other surgical risk-related systemic conditions
- Not enough information regarding the topic
- Animal or in vitro studies
- Articles published prior to 1 February 2009
- No access to the title and abstract

### 4.8. Strategy for Collecting Data

After the first literature analysis, the entire manuscript titles list was highlighted to exclude irrelevant publications, case reports and the non-English language publications. Then, research was excluded based on data obtained from screening just the abstracts. The final selection was performed reading the full texts of the papers in order to approve each study’s eligibility, based on the inclusion and exclusion criteria.

### 4.9. Record of the Extracted and Collected Data Extraction

The results and conclusions of the selected full text papers were used for assembling the data, according to the aims and themes of the present revision, as listed onwards.

The following parameters were used as a method for assembling the data and then organized following the schemes as seen in Table 1:

- “Author”—revealed the first author of publication
- “Year”—Year of publication
- “Type of study”—indicated the method of the research and some additional information
- “Sample size”—described the number of patients, animals or models examined
- “Protocols”—described types of groups or antibiotic protocols used

### 4.10. Risk of Bias Assessment

The grade of bias risk was independently considered, as reported in literature [43,44,45,46].

Potential causes of bias were investigated:

- Selection bias
- Performance bias and detection bias
- Attrition bias
- Reporting bias
- Examiner blinding, examiner calibration, standardized follow-up description, standardized residual graft measurement, standardized radiographic assessment

### 4.11. Third Molar Surgery

Octaves, if they break out correctly, can contribute to chewing and do not cause any problems as long as proper oral hygiene is applied. However, the fact that growth is subject to variations and imperfections is also due to the evolutionary course of the human species: in the past man needed more molars to chew raw and difficult foods, which were abandoned during evolution, while the dimensions of the mandible and jaw were clearly reduced, leaving little space for the normal development of the eighth tooth. Therefore, wisdom teeth are a necessity of the past. It is only with age that the jaws can reach a sufficient size to allow them to develop. Nevertheless, according to some anthropologists, the appearance of the eighth teeth in later age would have the task of counteracting the excessive wear of the other teeth. The prehistoric diet and the non-existence of dental care caused early tooth loss. In this way, the third molar, having sufficient space to develop, performed a reserve function, preserving correct chewing [47,48,49,50,51,52,53,54,55]. The avulsion of wisdom teeth may be a difficult surgery based on the local condition of the teeth and some patient-related factors. Surely, first of all, having a healthy and cooperative patient can make extraction easier. Depending on the degree of eruption of the dental element, the extraction can also be more or less simple. Surgical extraction of an erupted tooth in the arch could be simpler than that of teeth completely impacted in the maxillary bones or partially included in the mucosa. The first phase is always to practice a correct disinfection of the operating field, the oral environment always presents a bacterial charge and an inflammatory stimulus. The next phase involves the practice of proper local or regional anesthesia. Therefore, depending on the condition of the tooth, a surgical flap, osteotomy, odontotomy, and subsequently avulsion of the tooth element and any attached osteolytic lesion are performed. Once the extraction has been carried out, the flaps will be sutured. Certainly some anatomical variants, such as particularly long or curved roots and proximity to the mandibular canal, can make the extraction for the oral surgeon more complex [12,49,50,56,57,58,59,60,61,62,63,64,65,66,67].

### 4.12. Used Antibiotics in Dentistry and Antibiotics Properties

The use of antibiotics in dentistry, and more, is a fundamental clinical practice in eradicating bacterial infections. Therefore, the pathologies of dental expertise and, in particular, those of an endodontic nature are no exception. Typically, these are polymicrobial conditions involving Gram+ and Gram− bacteria, facultative anaerobes and narrow anaerobes. Antibiotics are actually the second most common pharmacological class prescribed by dentists, after painkillers. It is estimated that 10% of antibiotics are administered for dental needs (Table 4). Even the bacteria responsible for the most important dental infections, however, show signatures of tolerance to some classes of antibiotics, with the possibility that real forms of resistance may arise. In recent years there has been a growing awareness of antibiotic resistance. Despite this, several authors still show perplexity; data show that antibiotic administration is often excessive. Returning to the microbiological characteristics of endodontic infections, it should be emphasized that systemic antibiotic therapy is a fundamental adjuvant and not an alternative to orthodontic endodontic therapy, even more so in cases where the patient is not equipped with immune defenses adequate to contain the infection. The administration must therefore fall within scientifically determined and registered logics. The most widely used molecule is generally amoxicillin, alone or in association with clavulanic acid. Furthermore, in dental surgery it is also possible to mention topical intraoral antiseptics, among which we recognize different molecules, often used before, during and after surgical operations. Chlorhexidine is one of these, but also triclosan and povidone iodide are useful in breaking down the intraoral bacterial charge. Antibiotic protocols are used in dentistry not only for octave surgery, but also for other oral surgery surgeries. For example, all oral surgery interventions involving soft tissue injuries, such as fibromas or lipomas, are very common. Often the lesions can originate from a previous inflammatory state of the soft tissues, such as gingivitis and periodontitis [30,57,68,69,70,71,72,73,74,75,76,77,78,79,80]. Not all the methods to carry out diagnosis and epidemiological data collection are always efficient and it is not always possible, if there is no correct follow up, to carry out appropriate measurements of post-operative conditions [81]. It is interesting how different protocols for the same drug emerge from the works taken into consideration. Amoxicillin clavulanate is also often used for prophylaxis against bacterial endocarditis. The posologies have been made explicit in Table 4. It is difficult to highly differences between active ingredients, or even differentiate between the use and the non-use of antibiotics in others; it is even more complex to highlight clinical differences between different dosages of the same drug.

## 5. Conclusions

This study aimed to highlight the most widely used antibiotic protocols in dentistry, particularly during wisdom tooth surgery. The ultimate goal was to highlight any factors for or against one therapy over another. The population of clinicians is currently in favor of amoxicillin as evidenced in the results, although the real need to undertake an antibiotic protocol is still much debated in the literature. The use of antibiotics can involve some systemic adverse effects for patients and is not justified by literature. It is important to underline that performing an extraction of a third molar in the case of acute, chronic inflammation or in any case purulent infection without the use of antibiotics is not a topic often considered and taken into consideration. It must be considered that if wisdom teeth were extracted for orthodontic reasons and showed no inflammation, this could strongly influence the need for antibiotic therapy. Between the lines of the different articles taken into consideration, some assessed the surgeon’s experience and the speed or the duration of the intervention. Apparently, this factor is much more important and related to postoperative sequelae than the used drug therapy. Certainly, this study highlights and revises numerous articles, highlighting results in favor of antibiotic protocols. Still, there are few studies that support the non-use of antibiotic protocols during this surgery. Over time we will be able to evaluate these results by finding the most advantageous protocol for all the patients that need this kind of surgery.

## Figures and Tables

**Table 1:** Study selection and characteristics.

Author	Year	Type of Study	Sample Size	Protocols
Monaco et al. [13]	2009	RCT	59	Amoxicillin vs. placebo
Luaces-Rey et al. [14]	2010	RCT	160	Two amoxicillin different protocols
Siddiqi et al. [15]	2010	RCT, split mouth	100	Amoxicillin vs. placebo
Bezerra et al. [16]	2011	RCT, split mouth	800	Amoxicillin vs. placebo
Adde et al. [17]	2012	RCT	71	Amoxicillin vs. clindamycin vs. placebo
Sisalli et al. [18]	2012	RCT	107	Amoxicillin clavulanate vs. ceftazidime
Duvall et al. [19]	2013	RCT	30	Chlorhexidine 0.12% rinse vs. amoxicillin vs. placebo
Crincoli et al. [20]	2014	RCT, split mouth	24	Amoxicillin clavulanate vs. cefazolin
Arteagoitia et al. [21]	2015	RCT	118	Amoxicillin vs. placebo
Milani et al. [22]	2015	RCT	80	Two different routes of amoxicillin vs. placebo
Xue et al. [23]	2015	RCT, slit mouth	207	Amoxicillin vs. placebo
Braimah et al. [24]	2017	RCT	135	Two different routes of amoxicillin vs. levofloxacin

**Table 2:** Risk of bias table.

Risk of Bias	[13]	[14]	[15]	[16]	[17]	[18]	[19]	[20]	[21]	[22]	[23]	[24]
Low Risk		✓	✓	✓			✓			✓	✓	
Medium Risk								✓				
High Risk												
Unclear Risk	✓				✓	✓			✓			

**Table 3:** Evidence from the studies.

Author	Drug Side Effects	Quality of Life	Dysphagia	Trismus and Mouth Opening	Pain	Swelling	Fever	Wound Infection	Purulent Discharge	Edema and Abscess	Bacteremia	Lympha Deno Pathy
Monaco et al.					NS	NS	NS	NS				
Luaces-Rey et al.					NS	NS		NS	NS	NS		
Siddiqi et al.				NS	NS *p* >0.05	NS *p* >0.05	NS	NS	NS	NS		
Bezerra et al.				NS	NS *p* = 0.21			NS		NS		
Adde et al.					NS	NS *p* >0.05						
Sisalli et al.	NS				NS	NS		NS		NS		
Duvall et al.											NS	
Crincoli et al.			NS	NS	NS		NS			NS		NS
Arteagoitia et al.				S	S			NS		S		
Milani et al.				NS *p* = 0.99	NS	NS				NS *p* = 0.06		
Xue et al.					S *p* = 0.005			NS				
Braimah et al.		S										

S: significant; NS: Not significant.

**Table 4:** Protocols involving amoxicillin [69].

Active Principle Formulation	Before Surgery	After Surgery
Amoxicillin	2 g 1 h before surgery	500 mg every 8 h after surgery for 7 days
Amoxicillin clavulanate	500 + 125 mg 2 days before surgery	500 + 125 mg every 12 h for another 4 days.
Amoxicillin clavulanate	875 + 125 mg 2 days before surgery	875 + 125 mg every 12 h for another 4 days.
